# MicroRNAs/LncRNAs Modulate MDSCs in Tumor Microenvironment

**DOI:** 10.3389/fonc.2022.772351

**Published:** 2022-03-14

**Authors:** Xiaocui Liu, Shang Zhao, Hongshu Sui, Hui Liu, Minhua Yao, Yanping Su, Peng Qu

**Affiliations:** ^1^ Department of Histology and Embryology, Shandong First Medical University & Shandong Academy of Medical Sciences, Shandong, China; ^2^ Department of Pathophysiology, Shandong First Medical University & Shandong Academy of Medical Sciences, Shandong, China; ^3^ National Institutes of Health (NIH), Bethesda, MD, United States

**Keywords:** myeloid-derived suppressor cells, microRNAs, lncRNAs, networks, tumor microenvionment

## Abstract

Myeloid-derived suppressor cells (MDSCs) are a heterogeneous group of immature cells derived from bone marrow that play critical immunosuppressive functions in the tumor microenvironment (TME), promoting cancer progression. According to base length, Non-coding RNAs (ncRNAs) are mainly divided into: microRNAs (miRNAs), lncRNAs, snRNAs and CircRNAs. Both miRNA and lncRNA are transcribed by RNA polymerase II, and they play an important role in gene expression under both physiological and pathological conditions. The increasing data have shown that MiRNAs/LncRNAs regulate MDSCs within TME, becoming one of potential breakthrough points at the investigation and treatment of cancer. Therefore, we summarize how miRNAs/lncRNAs mediate the differentiation, expansion and immunosuppressive function of tumor MDSCs in TME. We will then focus on the regulatory mechanisms of exosomal MicroRNAs/LncRNAs on tumor MDSCs. Finally, we will discuss how the interaction of miRNAs/lncRNAs modulates tumor MDSCs.

## Introduction

MDSCs are a heterogeneous population derived from bone marrow progenitor cells and immature myeloid cells (1). In normal physiology, immature myeloid cells are differentiated into monocytes, granulocytes, macrophages and dendritic cells, which exert immune activity (2). However, in cancers and other diseases (such as inflammation), MDSCs have the negative regulatory immune response to exacerbate disease status (2, 3). In the process of tumor progression, MDSCs cannot properly differentiated into monocytes and macrophages to play their immune roles, but abnormally proliferate and accumulate within TME (4). Tumor cells secrete many factors to inhibit the differentiation of immature myeloid cells and promote the proliferation and immunosuppressive roles of MDSCs (5). These mediators mainly include the TGF-β, Ligands for toll-like receptors, IL-1β, IFN-γ, IL-6, FMS-like tyrosine kinase 3 ligand (FLT3L), Granulocyte colony-stimulating factor (G-SCF), Macrophage colony-stimulating factor (M-CSF), Granulocyte-macrophage colony stimulating factor (GM-CSF) and IL-4 (6). Most mediators activate the functional state of MDSCs by regulating signal converters and transcriptional activators (STATs and NFκB) (7, 8). For example, IL-6 enhances both stimulatory and inhibitory roles of MDSCs *via* STAT3 signaling pathways in breast cancer (8, 9).

Tumor MDSCs are mainly divided into two main subtypes according to their phenotypes and origins, which are defined by cell surface markers of MDSCs in both tumor models and cancer patients (10, 11). The phenotypes of MDSCs in tumor-bearing mice are defined using Gr1 (ly6G/ly6C)/CD11b and further include two subtypes of MDSCs: Monocyte-MDSCs (M-MDSCs, CD11b+Ly6G−Ly6Chi) and Granulocyte-MDSCs (G-MDSCs, CD11b+Ly6G+Ly6Clo) (12, 13). The phenotypes of MDSCs are more diverse in cancer patients. MDSCs are cell populations expressing Lin-HLA-DR-CD33+ or CD11b-CD14-CD33+ in human body. The main subtypes are also divided into M-MDSCs (HLA-DR−/loCD11b+CD14+ CD15−) and G-MDSCs (CD11b+ CD14− CD15+ or CD11b+CD14− CD66b+) (14). Third subtype known as early-MDSCs which has been found in human studies. They are defined as Lin-HLA-DR-CD33+, mainly consisting of colony-forming cells activity and other myeloid precursor cells (13, 15).

**Figure 1:**
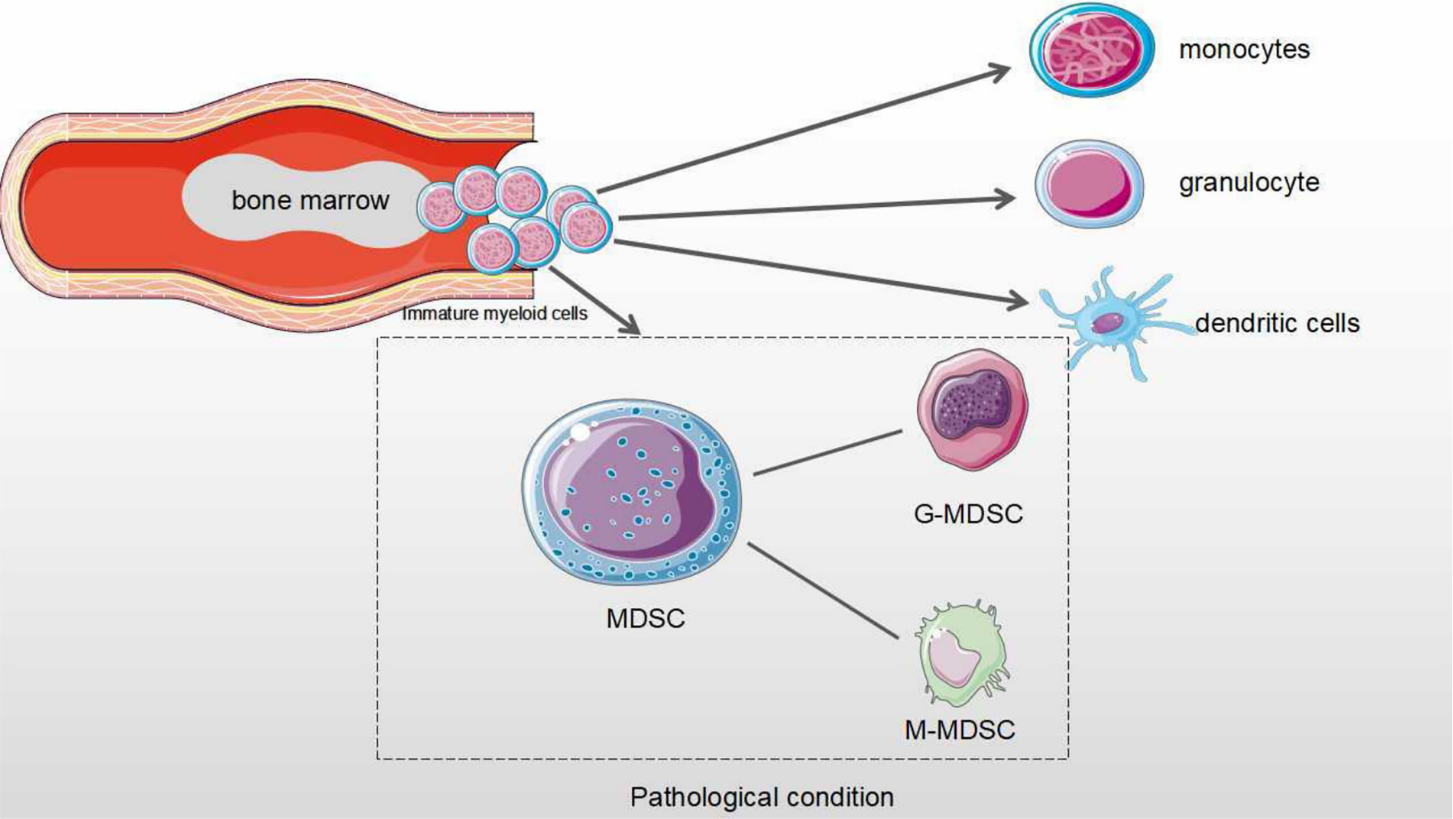
The differentiation process of MDSCs in physiological and pathological conditions. Under normal physiological conditions, myeloid progenitor cells are differentiated into monocytes, granulocytes and dendritic cells that are involved in the regulation of immune response. Under pathological condition, myeloid progenitor cells are differentiated into MDSCs. MDSCs from Immature myeloid cells are divided as two subtypes: monocytic MDSCs (M-MDSCs) and Granulocytic MDSCs (G-MDSCs).

M-MDSCs account for about 80% of all tumor MDSCs, but their inhibitory roles are lower than those of G-MDSCs. M-MDSCs are modulated by producing NO and Arginases. In contrast, the roles of G-MDSCs are determined by ROS and H2O2 (16–19). The main function of G-MDSCs is to inhibit T cell function, while M-MDSCs mainly differentiate into TAMs in cancer. It is well known that MDSCs have become the most important prognostic markers in cancer immunotherapy and contribute to immunosuppressive checkpoint resistance (20) (**Figure 1**).

MiRNAs are non-coding single-stranded small RNAs of approximately 22-24 nucleotides in length and are highly conserved evolutionarily, and are widely found in eukaryotic cells. They play vital regulatory roles in cells, especially in mRNA post-transcriptional regulation, and reduce mRNA expression levels by binding to the 3’UTR of mRNA and binding to the 5 ‘-UTR of mRNA to upregulate its transcription (10–12). MiRNAs are involved in regulating both a wide range of physiological activities such as cell cycle, differentiation, proliferation, maturation and immune response and pathological processes, such as inflammation and cancer (13). For example, our data have shown that miRNAs mediate the differentiation, expansion and function of tumor MDSCs (14).

**Figure 2:**
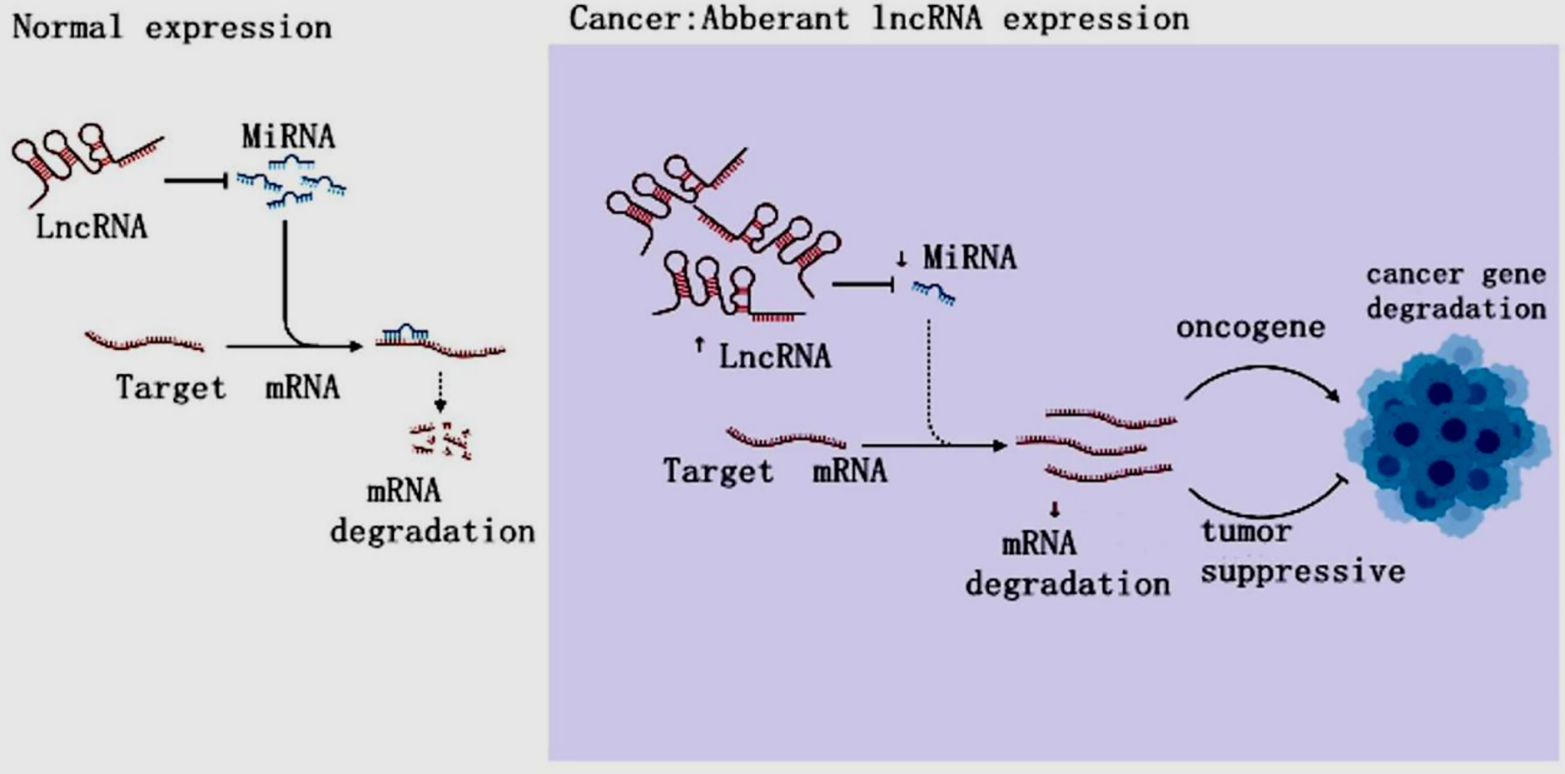
The LncRNA Regulation in cancer. In cancer, lncRNAs inhibit targeted mRNAs through endogenous competition with miRNAs, resulting in mRNA downregulation and carcinogenesis, resulting in tumor gene disorder and cancer.

LncRNAs are non-protein-coding RNAs of approximately 200 nucleotides in length (15). According to the position of lncRNAs in the genome relative to protein-coding genes, they can be divided into five categories: sense, antisense, bidirectional, intronic and intergenic (16, 17). LncRNAs are ever regarded byproducts of RNA polymerase II transcription as “noise” of genomic transcription without biological function (5, 17). However, the increasing evidences have revealed that LncRNAs mediate gene expression through chromatin modification, transcriptional regulation and post-transcriptional regulation in the nucleus and extranuclear, and are also involved in the occurrence and development of tumors (18–22) (**Figure 2**). LncRNAs have been found to mediate the carcinogenesis of colon cancer through a variety of molecular mechanisms, suggesting that lncRNAs can be used as biomarkers for early diagnosis and treatment of colon cancer (23). LncRNAs are overexpressed during the development, differentiation and activation of immune cells, such as monocytes, macrophages, dendritic cells, neutrophils (24). Furthermore, the increasing data were conducted on the activity of lncRNAs on MDSCs in TME (16, 25–27). Both miRNA and lncRNAs, as Non-coding RNAs (ncRNAs) can modulate tumor MDSCs. Thus, here we discuss the regulatory mechanisms of miRNAs/lncRNAs on the biological status and immune activity of MDSCs in TME, and put forward our own opinions.

## MiRNAs/LncRNAs

**Figure 3:**
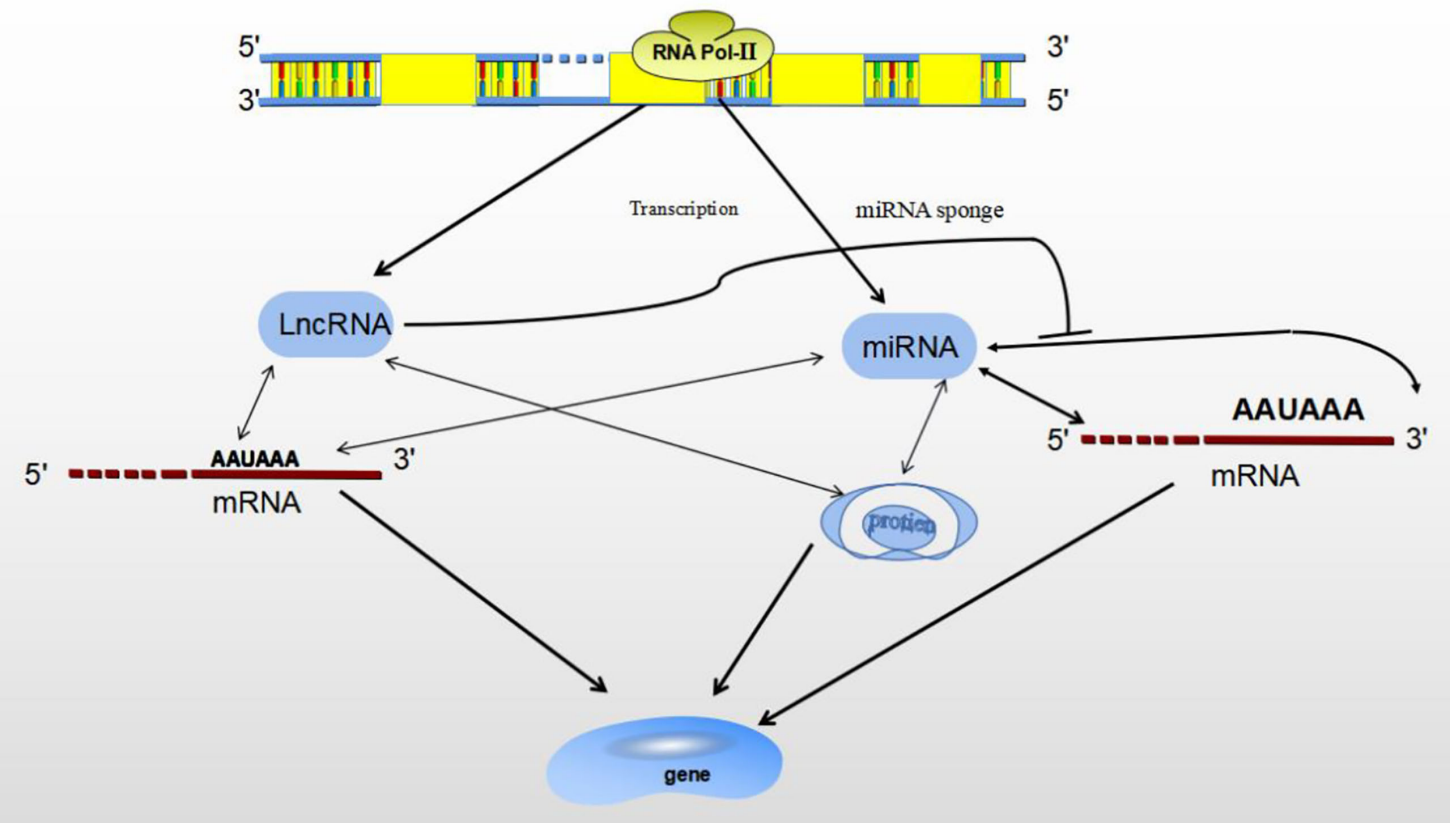
The relationship between MiRNA and LncRNA. Both miRNA and lncRNA regulate gene expression through binding with mRNA, gene/transcript or histone modifiers. LncRNA may sponges miRNA by base-pair blocking of miRNA response elements binding to target mRNA-3’UTR.

ncRNAs are mainly divided by length into small (< 200 nucleotides) and long (> 200 nucleotides) RNAs according to base length. ncRNAs are: miRNAs、lncRNAs、snRNAs、circRNAs (28). ncRNAs act as regulatory molecules that regulate for a wide range of cellular processes, such as chromatin remodeling, transcription and post-transcriptional modification (29). MiRNAs have been well investigated over the past decade, lncRNAs are actively studied for their diverse roles in gene expression regulation. Besides, lncRNAs themselves can interact with other ncRNAs, such as miRNAs (30). Both miRNA and lncRNA are transcribed by RNA polymerase II, and they play important roles in gene expression under both physiological and pathological conditions, as transcriptional and post-transcriptional regulators (28). Studies have shown that miRNAs and lncRNAs are involved in transcriptional regulation at different levels, miRNAs/lncRNAs directly determine gene expression by binding with mRNA, gene/transcript or histone modifiers (31). LncRNAs may play a functional role as miRNA sponges by base-pair blocking of miRNA binding to target mRNA-3’UTR (32) (**Figure 3**).

Recent studies have highlighted the diverse roles of MiRNAs/LncRNAs in cancer progression and metastasis. Increasing numbers of miRNAs and lncRNAs are found to be dysregulated in cervical cancer, regulating metastasis through regulating metastasis-related genes and signaling pathways. Moreover, miRNAs can interact with lncRNAs respectively during this complex process (33, 34). In breast cancer, the lncRNA MALAT1 and miR-100 are indirectly interlinked through VEGFA. MALAT1 binds to miR-216b as a competing endogenous RNA to restore Pyridox(am)ine-5- phosphate Oxidase deficiency (PNPO) and promote cell proliferation, migration and invasion in breast cancer (33). Therefore, miRNAs/lncRNAs are involved in gene expression and transcriptional regulation. They also affect the development of cancer and regulate the expression of oncogenes and tumor suppressors in TME (28, 32, 35). Therefore, the regulation of miRNA/lncRNA is more conducive to the research of bioactive targets for cancer treatment.

## Post-Transcriptional Regulation of Tumor MDSCs With MiRNAs/LncRNA

Researchers have found that the interactions between miRNAs/LncRNAs and transcription factor modulated the biological status and immune activity of MDSCs in TME (36). Here, we describe the regulation of miRNA/lncRNA on MDSCs in the TME. The abnormal expression of miRNAs/lncRNAs in MDSCs and their regulatory mechanism on MDSCs have become potential breakthrough points.

### Expansion of MDSCs

**Figure 4:**
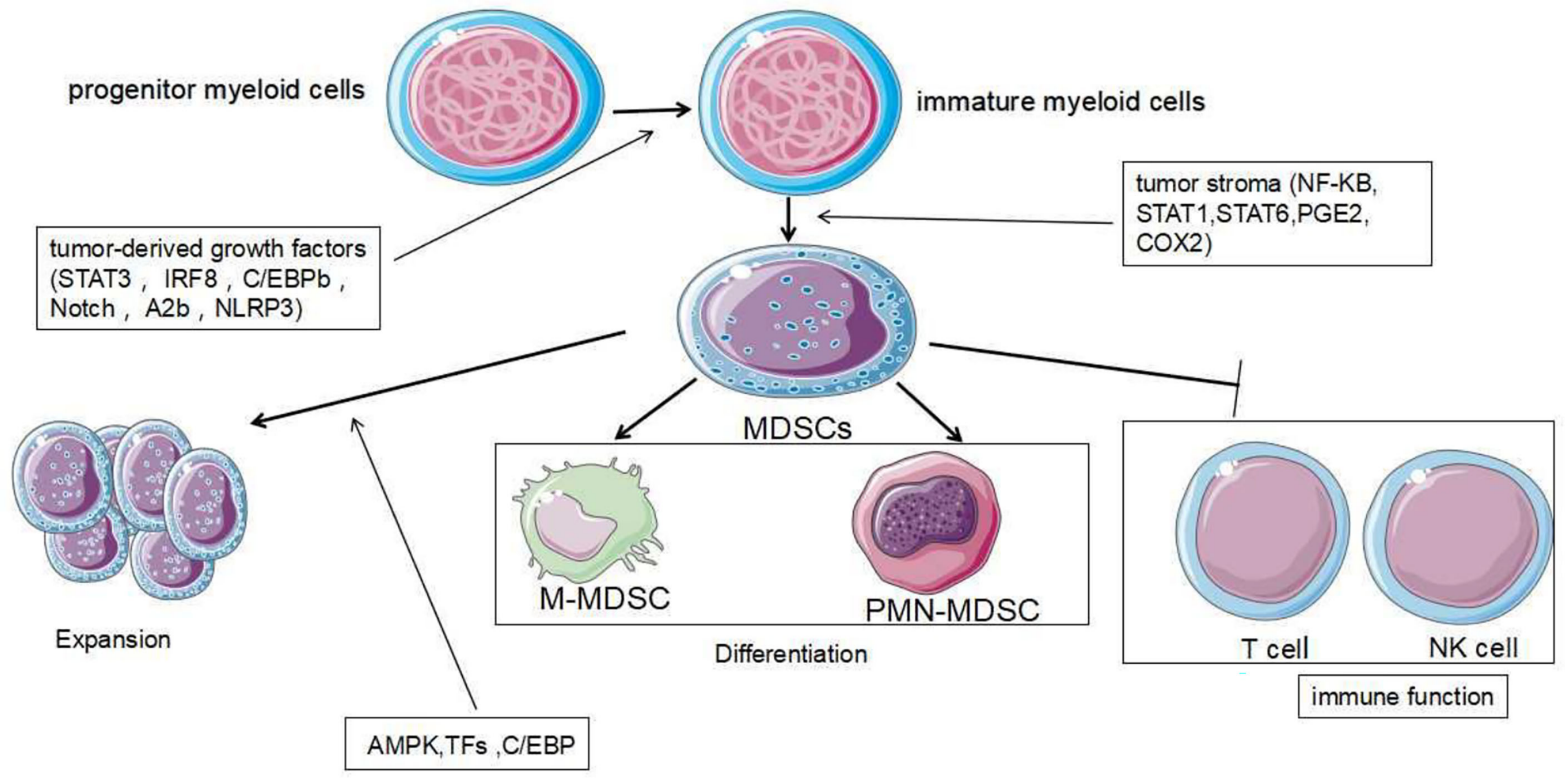
Development and role of MDSCs. MDSCs are differentiated from myeloid progenitor cells. During the differentiation process, two signaling models are mainly used: The signaling driven by tumor-derived growth factors (STAT3, IRF8, C/EBPb, Notch and NLRP3) is responsible for proliferation of immature bone marrow cells and inhibits their differentiation. The second type of signaling is mediated by factors which are produced by tumor stroma (NF-KB, STAT1, STAT6). It is responsible for pathological development of immature myeloid cells into MDSCs. The expansion and immune function of MDSCs are regulated by other different signaling mechanisms further.

The expansion of tumor MDSCs is regulated through several pathways. Members of the CCAAT/enhancer binding protein (C/EBP) family, as key regulatory transcription factors, may regulate many biological processes, including cell growth, differentiation, metabolism and death. In TME, C/EBP maintains the critical regulation of MDSCs (37, 38) (**Figure 4**). In Lewis lung carcinoma and B16 melanoma, the overexpression of miR-486 promotes the proliferation of MDSCs and inhibits the differentiation and apoptosis of MDSCs through targeting C/EBPA (39). During the tumor process, when the C-X-C motif chemokine receptor 2(CXCR2) is activated, the expression level of miR-449c targeting STAT6 mRNA in MDSCs is upgraded to promote the MDSC expansion (6). In 4T1-breast cancer cell, miRNA-494 which is upregulated by tumor-derived factor TGF-β1, promotes the accumulation and activity of MDSCs through targeting Phosphatase and tensin homolog (PTEN) and activating Akt pathway (40). miR-155 and miR-21 promote the expansion of tumor MDSCs through targeting ship-1 and PTEN (41). Furthermore, miR-155 enhances tumor MDSC inhibitory activity through SocS1 repression (42–44). MiR-155 deficiency is also found to diminish the aggregation of functional MDSCs in the colon cancer, indicating that miRNA-155 could accelerate the accumulation of MDSCs (43–45). In lung tumor mouse model, miR-21 maintained MDSC accumulation in the TME by downregulating RUNX1 and upregulating Yes-associated protein (YAP), indicating that targeting miR-21 in MDSCs may be developed as an immunotherapeutic approach to combat lung cancer (46). In mixed leukemia, tumor-secreted factors GM-CSF/IL-6 upregulate high expression levels of miR-21a/21b/181b through STAT3/CEBPβ pathway, further diminishing the expression of WD repeat-containing protein 5 (Wdr5), absent small or homeotic-like (ASH2L) and mixed lineage leukemia 1 (MLL1), which are involved in the expansion and differentiation of G-MDSC. Furthermore, knockdown of these miRNAs diminishes the expansion of GM-CSF/IL-6-induced G-MDSCs, suggesting that miR-21a/21b/181b stimulate accumulation of MDSCs in the TME (47) (**Table 1**).

**Table 1:** Regulation of MiRNAs on tumor MDSCs.

MiRNA	Targets/signal pathway	Function on MDSCs	Ref.
miR-449c	STAT6	To elevate the number	(6)
miR-142-3p	STAT3/CEBPβ	To prevent the differentiation	(14, 36, 48)
miR-223	MEF2C	To block accumulation/differentiation	(39)
miR-486	C/EBPα	To stimulate proliferation	(40)
miR-494	PTEN/Akt	To promote the accumulation and activity	(49)
	TGF-β	To strengthen function	(41)
miR-155	PTEN	To increase the number	(41)
miR-21	AMKP	To exaggerate expansion	(47)
miR21a/21b/181b	AMPK	To maintain inhibitory roles	(50)
miR-34a	MUC1	To diminish the expansion	(51)
	TGF-β/IL10	To reduce the number	(52)
miR-10	STAT3/CEBPB	To stimulate expansion	(53, 54)
miR-708	RaP1B	To diminish the expansion and number	(55)
miR-424		To reduce numbers	(56)
miR-9	NFIA	To improve differentiation	(57)
miR-136		To promote differentiation	(58)
miR21/miR130b/miR155/miR28	IGF1/Jun	To enhance blockage activity	(59)
miR-200c		To enhancing inhibitory activity	(54)
miR-17-92	FOG2/PTEN	To diminish blockage roles	(60)
miR-195/miR-16	STAT	To block immunosuppressive function	(61)
miR-10a	PD-l	To improve the differentiation	(62)
miR-30a	Runx1	To enhance activity	(63)
miR-17	SOCS3	To prevent differentiation/activity	

Chemotherapy is one major method of cancer treatment. However, it also brings some side effects. Rong et al. found that chemotherapies (such as doxorubicin treatment) induced drug-resistance in breast cancers cells and stimulated proliferation and activation of MDSCs to inhibit T cell anti-tumor response. In doxorubicin-resistant breast cancer, Doxorubicin-induced miR-10 overexpression exaggerates the expansion and activation of MDSCs by activating the AMKP signaling pathway, leading to poor prognosis in breast cancer patients (52). Hox antisense intergenic RNA (HOTAIR) is one lncRNA which is regarded as oncogene to play crucial roles in the progression and metastasis of several cancers such as breast, colorectal and gastric cancers. Moreover, HOTAIR overexpression causes expansion and recruitment of MDSCs in cancer cells through the release of CCL2 (5) (**Table 2**).

**Table 2:** Regulation of LncRNAs on MDSCs.

LncRNAs	Target genes/signal pathway	Function on MDSCs	Ref.
HOTAIR	CCL2	To promote expansion and recruitment	(5)
RNCR3	miR-185 CHOP	To increases the inhibitory roles	(16)
Olfr29-ps1	IL-6	To accelerate roles	(16)
lnc-C/eBPβ	C/EBPβ/WDR5/IL-4il	To improve the differentiation	(47)
	C/EBPβ subtypes LAP	To suppress immunosuppressive function	(46)
LNC-CHOP	CHOP and C/EBPb	To improve blockage the roles	(64)
lncRNAPVT1	hypoxia-inducible factor-1α	To upgrade the inhibitory activity	(65)
AK036396	Ficolin B	To prevent the maturation	(66)
lncR MALAT1		To decrease the number	(67)

**Figure 5:**
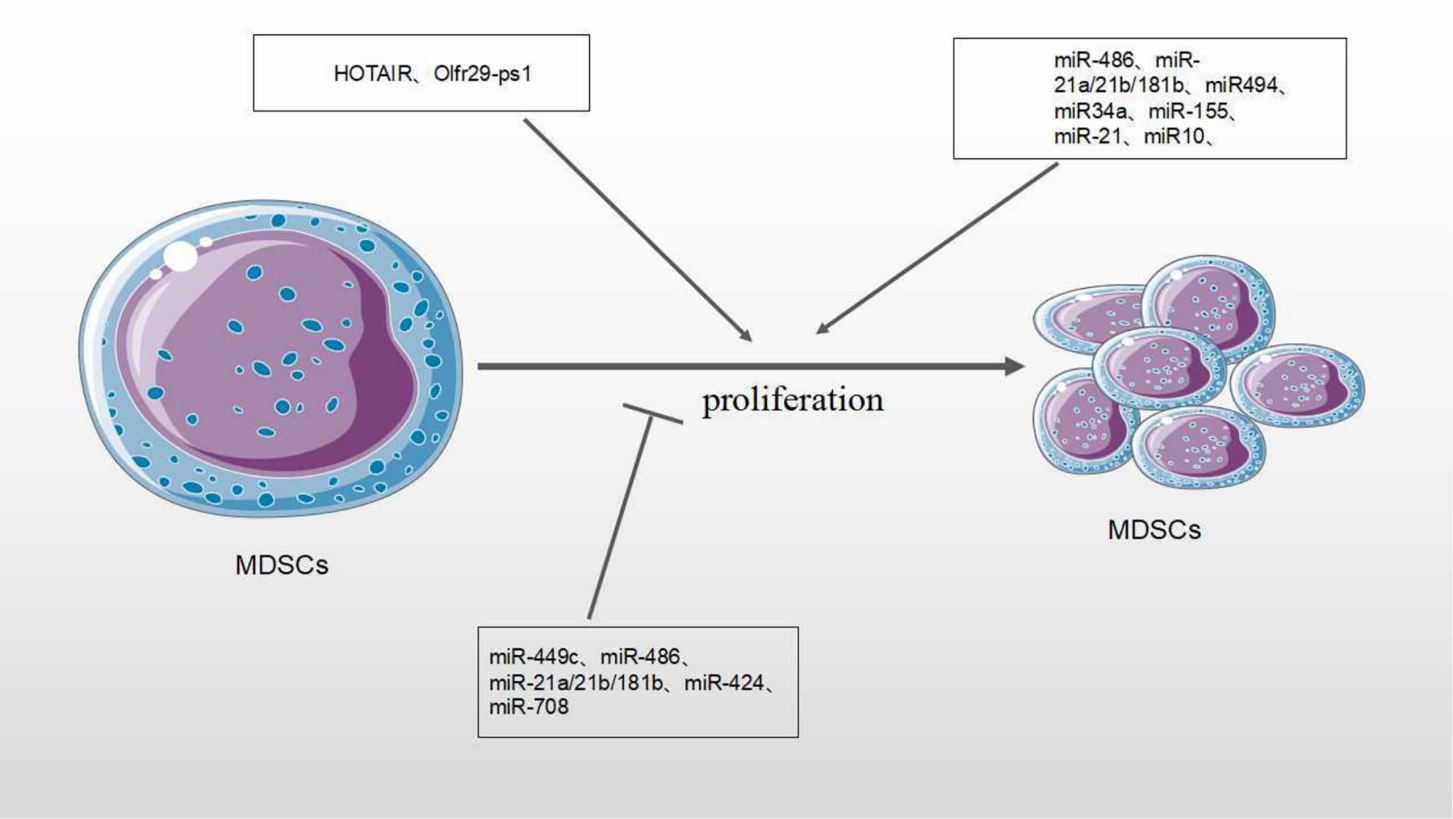
Effect of MicroRNA/LncRNA on MDSC’ proliferation in the TME. MiRNAs/LncRNAs modulate the proliferation of MDSCs through different genes and signaling pathways. In each process, microRNA/LncRNA play positive: ⊣ or negative: ⊣ roles.

MiRNAs/lncRNAs also negatively regulate the numbers and expansion of MDSCs in the TME. In our previous studies, negative roles of miRNAs on MDSCs have been described (14). In tumor-bearing mice, miR-223 reduces the accumulation of MDSCs and inhibits immature myeloid cells differentiation into MDSCs by targeting Myocyte enhancer factor 2C (MEF2C) (36, 48). In ovarian cancer treatment, Lin et al. found that dexamethasone(DEX), a synthetic glucocorticoid (GC), stimulated miR-708 overexpression by targeting RaP1B, further diminishing the expansion and number of MDSCs in TME (53). Pyzer et al. demonstrated that miR34a overexpression led to the downregulation of c-myc expression by transmembrane glycoprotein Mucin 1 (MUC1) silencing, reducing the expansion of MDSCs in acute myeloid leukemia (AML) (50) (**Figure 5**).

### Differentiation of MDSCs

Tumor-derived factors affect different stages of myeloid cell differentiation, leading to the generation of pathologically activated M-MDSCs and PMN-MDSCs. The differentiation process of MDSCs is mediated through two types of signaling panels. The first type of signaling, driven by tumor-derived growth factors (STAT3, IRF8, C/EBPb, Notch and NLRP3), is responsible for proliferation of immature bone marrow cells and inhibits their differentiation. The second type of signaling is mediated by factors produced by tumor stroma (the NF-KB Pathway, STAT1, STAT6). It is responsible for pathologically activating immature myeloid cells into MDSCs (68) (**Figure 4**). Accumulating evidence has demonstrated that tumor-related MDSCs are differentiated into mature myeloid cells, such as macrophages or neutrophils through the regulation of different miRNAs. The downregulation of miR-9 is found to improve the differentiation of tumor MDSCs *via* targeting Runx1, thereby hindering tumor growth (56). Shi et al. demonstrated that TNF-α-upregulated miR-136 enhanced the differentiation of MDSCs and inhibited tumor growth by targeting Nuclear factor I A(NFIA) (57).

MiRNAs/lncRNAs also negatively modulate the differentiation of MDSCs in the TME. The upregulation of miR-34a reduces immature myeloid cells differentiation into MDSCs *via* TGF-β and IL-10 (51). The productions of bone marrow are altered during tumor development, leading to the accumulation of immunosuppressive cells there. miR-142-3p is found to restrain the differentiation of MDSCs into mature cells by regulating STAT3 and C/EBPβ signaling pathways (13, 14). MiR-17 family members (such as miR-17-5p, miR-20a and miR-106a) are overexpressed in human progenitor cells and inhibit AML1(the leukemia-associated transcription factor acute myeloid leukemia 1; also known as runt-related transcription factor 1, or RUNX1), leading to downregulation of M-CSFR, which prevents differentiation and activity of tumor MDSCs (63). In human acute promyelocytic leukemia, the master transcription factor PU.1 is revealed to activate the transcription of miR-424 and repress NFI-A, an inhibitor of monocyte differentiation, thereby stimulating the differentiation of MDSCs into mature cells to reduce MDSC population (55) (**Table 1**).

**Figure 6:**
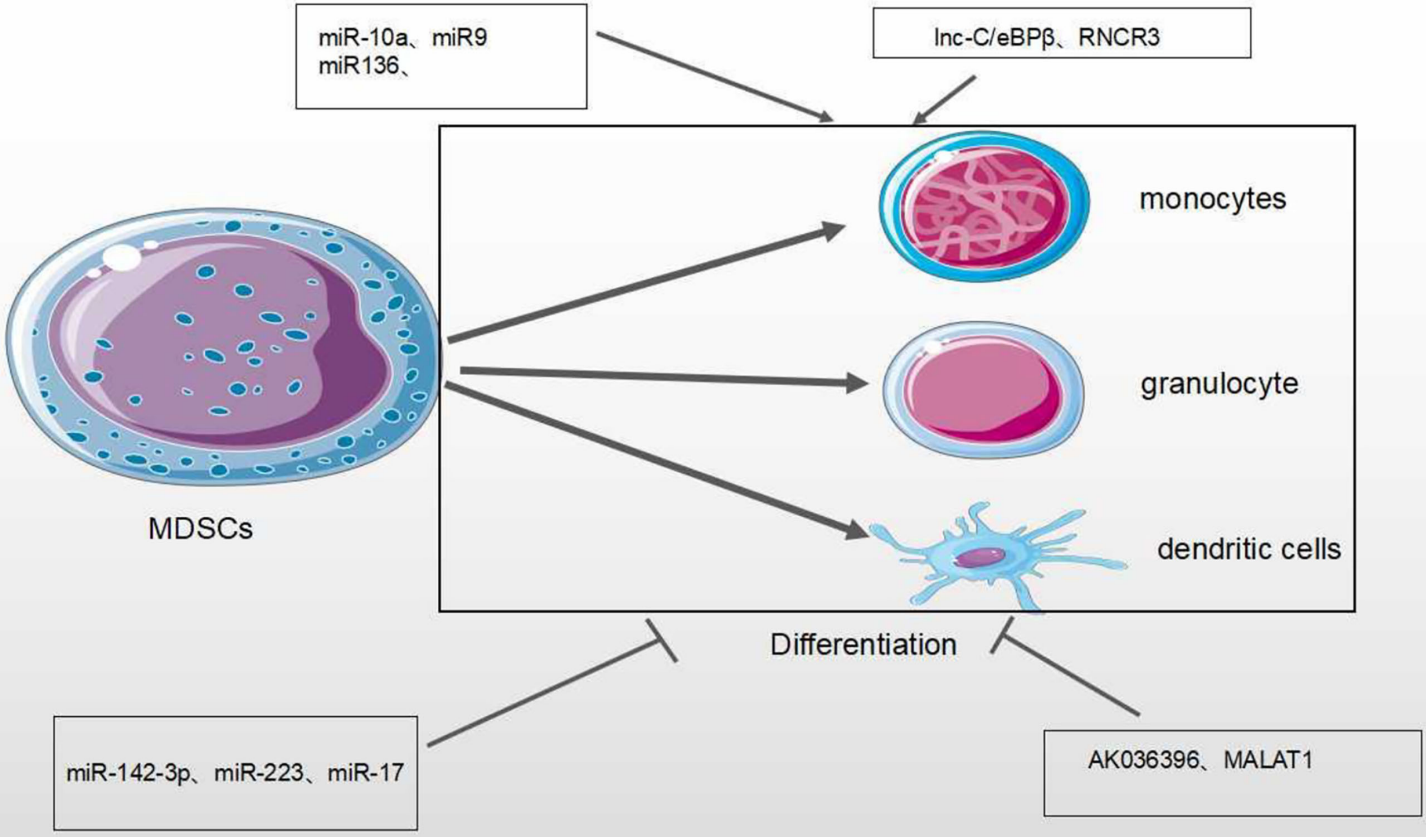
Effect of MicroRNA/LncRNA on MDSC’ differentiation in the TME. MiRNAs/LncRNAs mediate the differentiation of MDSCs into monocyte, dendritic cells and neutrophils through different genes and signaling pathways. In each process, microRNA/LncRNA play positive: ⊣ or negative: ⊣ roles.

Lnc-C/EBPβ is an intermediate gene encoded on chromosome 4 that is highly conserved in mice, humans and other species. There are two subtypes of c/EBPβ: liver-rich activating protein (LAP*, LAP) and liver-rich inhibitory protein (LIP) (64). Expression of lnc-C/EBPβ in murine M-MDSCs is found to block the differentiation and inhibitory activity of MDSCs. This is through down-regulating the expression of IL-4, suggesting that it could be a potential target in tumor immunotherapy (46, 47). Metastasis-Associated Lung Adenocarcinoma Transcript 1 (MALAT1), a nuclear intergenic lncRNA, is highly conserved among species and involved in various diseases. Recently, lncRNA MALAT1 was found to stimulate the proliferation, invasion, and metastasis of many types of cancer cells such as cervical cancer, lung cancer, colorectal cancer and liver cancer (69). Knockout of MALAT1 genes in MDSCs lead to the increased number of MDSCs by the inhibition of MDSC differentiation (67). However, the regulatory mechanisms need to be investigated further (**Figure 6** and **Table 2**).

### Immunosuppressive Function of MDSCs

In the TME, MDSCs inhibit the anti-tumor roles of many immune cells, such as Natural Killer (NK) cells, B cells and T cells. The inhibition of T cell function is most important for evaluating the activity of MDSCs (1) (**Figure 4**). MiRNAs/lncRNA upregulate the activity and immunosuppressive function of MDSCs through different signaling pathways and transcription factors within TME (39, 58). In B lymphoma mouse models, the expression of miR-30a in MDSCs promotes the immunosuppressive roles of MDSCs (56). In addition, miR-30a also targets SOCS3/STAT3 to enhance the inhibitory activity of MDSCs (55). In the most common type of non-Hodgkin lymphoma (NHL) —– diffuse large B-cell lymphoma (DLBCL), four circulating miRNAs (miR-21, miR-130b, miR-155, and miR-28) are considered to be novel prognosis biomarkers of DLBCL and modulate RAS protein signaling transduction *via* Insuline-like growth factor I(IGF1) and Jun. These four miRNAs are associated with the induction of MDSCs and Th17 cells through cytokines TGFB1, IL-6 and IL-17, resulting in the immune suppression of DLBCL (58). In gastric cancer, miR-494 is positively associated with the expression of tumor-derived TGF-β which exaggerates the suppressive roles of MDSCs (49). In various tumor mouse models (such as lung cancer, breast cancer and colon cancer), it has been found that the tumor-derived factor GM-CSF induces miR-200c overexpression to activate Akt by negatively regulating the transcriptional regulator friend of Gata 2 (FOG2) and PTEN expression, further enhancing the immunosuppressive activity of MDSCs (59) (**Table 1**).

Olfactory Receptor 29 Pseudogene 1 (Olfr29-ps1), as one lncRNA pseudogene, is conserved in vertebrates (70). Tumor-associated factors can increase the expression of Olfr29-ps1 in MDSCs. In colon and rectal cancer, Olfr29-ps1 stimulates proliferation and inhibitory activity of M-MDSC by the upregulation of pro-inflammatory factor IL-6 (16). Plasmacytoma Variant Translocation 1 (PVT1), an intergenic lncRNA, is conserved in humans and mice. In various cancers, tumor-associated factors induce the increased expression of PVT1 in MDSCs. Downregulation of Pvt1 expression in PMN-MDSCs can reduce suppressive activity of MDSCs through the reduced activity of both ROS and Arg-1. In addition, PVT1 also up-regulates the expression levels of hypoxia-inducible factor-1α to enhance the immunosuppressive activity of G-MDSCs under hypoxia (65). Similarly, lnc-CHOP, as an intronic lncRNA, increases the activity of both ROS and Arg-1 through interacting with both CHOP and C/EBPβ subtypes to promote C/EBPβ activity and H3K4me3 enrichment, further enhancing the suppressive activity of MDSCs within the TME (64).

Tian et al. found that lncRNA AK036396 and its target Ficolin B were highly expressed in mouse PMN-MDSCs. The downregulation of lncRNA AK036396 improved differentiation and diminished the suppressive roles of PMN-MDSCs through reduced Ficolin B protein stability. In addition, human M-ficolin, as an ortholog of mouse Ficolin B, stimulates the suppressive activity of MDSCs in patients with lung cancer through the induction of arginase1 expression. These results indicate that lncRNA AK036396 could accelerate inhibitory roles of PMN-MDSCs on T cell anti-tumor responses (66) (**Table 2**).

MiRNAs/lncRNAs also negatively modulate the immunosuppressive function of MDSCs in the TME. The STATs pathway is of vital regulatory function. In both lung carcinoma and 1D8 ovarian carcinoma, miR-17-92 cluster (miR-17-5p and miR-20a) could block the roles of MDSCs through targeting STATs (54). Tao et al. demonstrated that the restoration of miR-195 and miR-16 expression enhanced radiotherapy *via* T cell activation in TME by the inhibition of PD-L1 expression, after radiation with anti-PD-1 treatment on prostate cancer. The synergistic effect of immunotherapy and radiotherapy is associated with the proliferation of CD8+ T cells and inhibition of MDSCs and regulatory T cells (Treg), indicating that miR-195 and miR-16 may reduce the suppressive functions of MDSCs through PD-1 dependent pathways (60).

An intergenic lncRNA, HOXA Transcript Antisense RNA Myeloid-Specific1 (HOTAIRM 1) has been shown to downregulate the suppressive functions of MDSCs in the TME, since HOTAIRM1 can induce the high expression of HOXA1 in MDSCs to reduce Arg-1 expression and ROS production. In addition, increased expression of HOXA1 has been shown to decrease the percentage of MDSCs, and enhance the immune response in a tumor mouse model (26).

**Figure 7:**
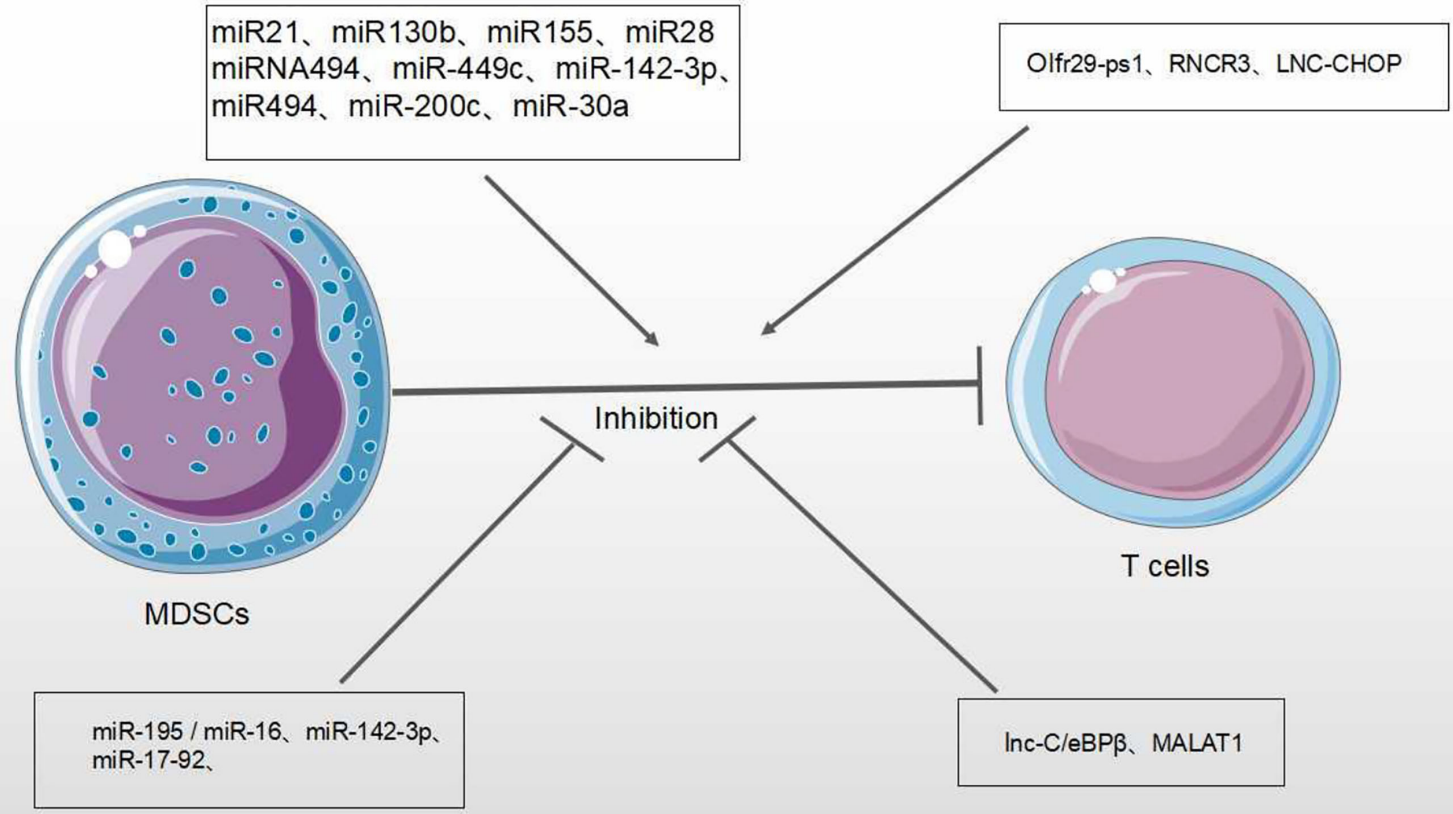
Effect of miRNA/LncRNA on MDSC’ function in the TME. MiRNAs/LncRNAs modulate the immunosuppressive roles of MDSCs on T cell anti-tumor response. In each process, miRNAs/LncRNAs play positive → or negative ⊣ roles.

Therefore, miRNAs/lncRNAs effectively regulate the differentiation, proliferation, and immunosuppressive functions of MDSCs (47, 71) (**Figure 7**).

## MiRNAs/LncRNAs From Tumor-Derived Exosomes Mediate the Function of MDSCs in TME

Exosomes are small extracellular vesicles with size 30-150nm in diameter that are secreted by most cells (72). Exosomes are rich in genetic material and molecules: DNA, miRNA, lncRNAs, proteins and lipids, which are essential for cell-cell communication and physiological status. Moreover, exosomes are also involved in the regulation of tumor progression in the TME (11). Recently, it has been demonstrated that exosomes secreted by tumor cells play critical roles in cancer progression and invasion, including TME remodeling, tumor metastasis and tumor-associated immunosuppression (73).

MiRNAs in tumor-derived exosomes mediate the activity of MDSCs in TME through different expression patterns, transcription factors and signaling pathways (61, 74). In pancreatic cancer, the increased expression of miR-let-7i in TDEs affects the levels of myeloid inhibitory intracellular inflammatory cytokines (IL-6, IL-17, IL-1β) and transcription factors, downregulating the anti-tumor immune response (74). In glioma, glioma-derived exosomes (GDEs) miR-29a and miR-92a increase the proliferation and suppressive roles of MDSCs through targeting high-mobility group box transcription factor 1 (Hbp1) and protein kinase cAMP-dependent type I regulatory subunit alpha (Prkar1a), respectively, further mediating the formation of suppressive TME (75). In LLC lung cancer model, miR-21a from LLC-Exosomes are revealed to increase both the autocrine production of IL-6 and phosphorylation levels of STAT3 by targeting Programmed cell death 4(PDCD4), thereby preventing the activation of cytotoxic CD8+T cells and enhancing the proliferation and activity of MDSCs (76). In addition, in hypoxia-induced GDEs miR-10a and miR-21 stimulate the expansion and activation of MDSCs by targeting RAR-related orphan receptor α (RORA) and PTEN (77). In breast cancer with high expression of interleukin-6, TDEs miR-9 and miR-181A activate the JAK/STAT to exaggerate the proliferation and inhibitory roles of MDSCs by targeting SOCS3 and PIAS3 (76). In gastric cancer, Ren et al. found that the TDE miR-107 prompted the proliferation and activation of MDSCs by targeting Dicer1 and PTEN (78) (**Table 3**).

**Table 3:** Tumor derived exosome miRNA on tumor MDSCs.

MiRNAs	Target genes/signal pathways	Function on MDSCs	Tumor	Ref.
miR-29a miR-92a	PDCD4/STAT3	To improve differentiation	Glioma	(75)
miR-10a/miR-21	Dicer1/PTEN	To enhance the expansion/activation	Glioma	(77)
miR-21a	SOCS3/PIAS3/JAK/STAT	To exaggerate the proliferation/immunosuppressive functions	LLC	(76)
miR-9/miR-181a	RORA/PTEN	To promote the proliferation and activity	Breast cancer	(76)
miR-107		To induce the proliferation/activation	Gastric cancer	(78)
miR-let-7i	IL-6/IL-17/IL-1b	To restrain the roles	Pancreatic cancer	(74)

lncRNAs are also secreted in exosomes as messengers of intercellular communication. Some lncRNAs are enriched in exosomes, while others are almost absent, suggesting that some lncrnas are selectively trafficked into exosomes. Furthermore, RNA sequencing in exosomes derived from tumors revealed that most of the non-coding transcripts of exosomes were lncRNAs (79). Meanwhile, exosomal LncRNAs are often found in clinical cancer samples, indicating that LncRNA may be a potential biomarker for cancer diagnosis. In primary urothelial bladder cancer (UBC) cells, exosomic lncRNA HOTAIR is secreted by proteins (SNAI1, TWIST1, ZEB1 and LAMB3), which regulate EMT, resulting in gene changes on epithelial cells. LncRNA ZFAS1 is found to increase in the serum exosomes of GC patients with gastric cancer (GC), suggesting that lncRNA ZFAS1 plays a positive role in the progression of gastric cancer (80). Exosomal LncRNA ZFAS1 also promotes the proliferation, migration and invasion of tumor cells from esophageal carcinoma (ESCC), and inhibits the apoptosis of ESCC cells by up-regulating STAT3 and down-regulating MiR-124, leading to the carcinogenesis of ESCC. LncRNA ZFAS1 is believed to be a competitive endogenous RNA regulating MiR-124, thereby enhancing STAT3 expression (81). In bladder cancer (BCs), lncRNA-PTENP1 is found to be reduced in tissues and plasma exosomes. Cells which secrete exosomal PTENP1, deliver it to BC cells to inhibit the biological malignant behavior of BC cells by increasing apoptosis and decreasing invasion and migration (82). The regulatory mechanism of TDEs lncRNAs on MDSCs has not been clarified thoroughly. A few studies have shown that TDEs lncRNAs play the important regulatory role in TME and tumor cell interactions, accelerating tumor growth (24). TDEs lncRNAs are transported to the TME to modulate the roles of various cells, including macrophages, endothelial cells and fibroblasts (24). In liver cancer cells, lncRNA TUC339 induces M2 polarization by interacting with cytokine-cytokine receptors to exaggerate tumor metastasis (83). It is well known that lncRNA urothelial carcinoma-associated (UCA1) is an lncRNA associated with the occurrence and progression of various cancers, including colorectal cancer. Meanwhile, the mechanism of tumor-derived exosome lncRNA-UCA1 has also been studied. In colon cancer (CRC), UCA1 plays a key role in CRC tumor progression by packaging into exosome, and UCA1 sequesters mir-143 *via* a sponge mechanism (84) (**Table 4**). However, the mechanism by which exosome miRNA/LncRNA affects MDSC in TME remains to be studied.

**Table 4:** Tumor derived exosome LncRNA on cancer.

LncRNAs	Target genes/signal pathways	Function on cancer	Tumor	Ref.
LncRNA HOTAIR	SNAI1/TWIST1/ZEB1/LAMB3	To changing epithelial cells	UBC	(80)
LncRNA ZFAS1		To promoting the cancer	Gastric cancer	(80)
LncRNA ZFAS1	STAT3/MiR-124	To promoting the proliferation, migration and invasion of tumor	Esophageal carcinoma	(81)
LncRNA-PTENP1	PTENP1	To inhibiting the biological malignant behavior of BC cells	Bladder cancer	(82)
LncRNA TUC339	M2 polarization	To exaggerating tumor metastasis	Liver cancer	(83)
LncRNA-UCA1	Mir-143	To promoting the cancer	Colon cancer	(84)

## LncRNA/MiRNA Interaction Regulate MDSCs in TME

**Figure 8:**
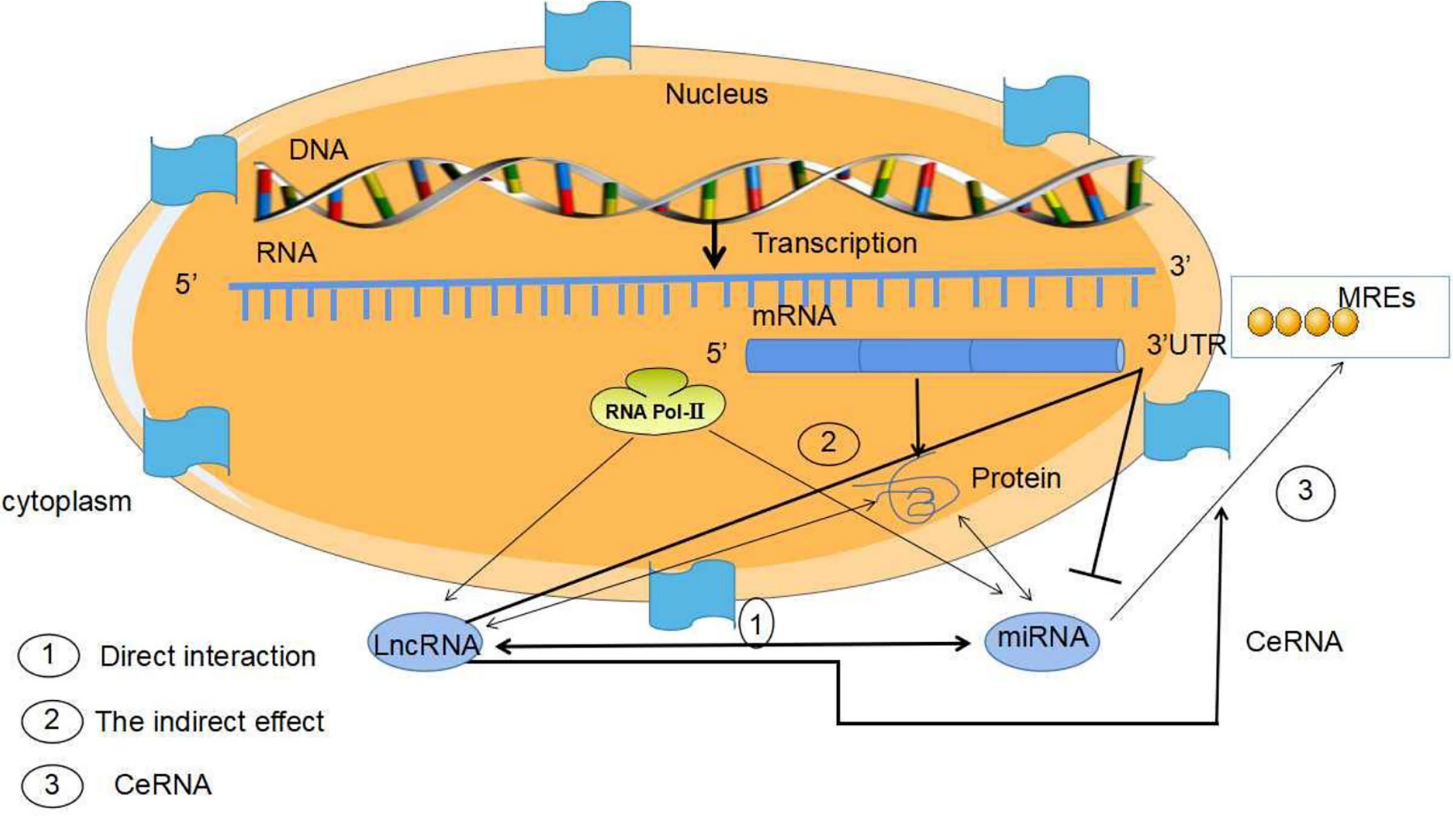
Mechanism of MiRNA/LncRNA interaction. The interaction mechanism between miRNAs and lncRNAs is as follows: (1) The two directly interact with each other. (2) lncRNAs inhibit miRNAs by competitively binding the 3 ‘-UTR of miRNA target mRNA. (3) lncRNAs, as ceRNA, inhibit the expression of miRNA by using the “miRNA sponge”. MiRNAs may regulate lncRNA expression through the similar mechanism by which miRNAs regulate mRNA, since LncRNA structure is similar to that of mRNA.

LncRNA not only directly participates in the regulation of gene expression, but also regulates the expression of miRNA (85). miRNA can regulate mRNA expression through the miRNA response elements (MREs) of mRNA 3 ‘- UTR. LncRNA can adsorb miRNA through MREs to competitively bind miRNA as one Competing endogenous RNA (ceRNA) and interfere with the binding of miRNA with downstream target genes, and then participate in various biological processes such as cell proliferation, differentiation, apoptosis and angiogenesis (86, 87). Luan et al. reported that LncRNA XLOC_006390z played a functional role as one ceRNA in cervical cancer. When XLOC_006390 is knocked out, the expression of Mir-331-3p target gene NRP2 and Mir-338-3p target gene PKM2 is significantly downregulated, further promoting the occurrence and metastasis of cervical cancer (88). MiRNA can regulate lncRNA expression as well as target mRNA expression. LncRNA structure is similar to mRNA. LncRNAs indirectly inhibit the negative regulation of miRNAs on target genes by competing with miRNA to bind the 3’-UTR of target gene mRNA. Some of lncRNAs can form miRNA precursors through intracellular shearing, and then process and generate specific miRNAs to regulate the expression of target genes and exert functions. In addition, Individual lncRNAs function as endogenous miRNA sponges and inhibit miRNA expression, further performing biological roles. Therefore, integrated analysis of the regulatory relationship between mirNA-lncrNA-mrna can explain the occurrence and development of diseases comprehensively. These indicted that miRNA may regulate lncRNA expression through the similar mechanism by which mRNA is regulated (**Figure 8**).

Mir-155 was overexpressed in MEGO1 leukemia cell line and the expression level of target lncRNA was significantly decreased. When Mir-155 was silenced, the expression of target lncRNA was significantly increased. These results indicated that miRNA could regulate the expression of lncRNA (89). lncRNAs can act as one ceRNA to sequester miRNAs, regulating the abundance and activity of miRNAs, resulting in the de-repression of genes targeted by corresponding miRNAs in cancer progression (34, 90). Recently, the regulation of lncRNA/miRNA in MDSCs has become increasingly important. Studies have speculated that lncRNA-miRNA may have synergistic effects on the roles of MDSCs. MiR-9 and or Runx1 overlapping RNA (RUNXOR) are two non-coding RNAs involved in the differentiation and activation of MDSCs. Tian et al. showed that miR-9 directly downregulated the expression of lncRNA Runx to stimulate the differentiation of MDSCs and reduce the suppressive ability of MDSCs (36). The retinal non-coding RNA3 (RNCR3), an intragenic lncRNA, which is conserved sequence in mammalian genomes, has been shown to be highly expressed in glioblastoma and prostate cancer (91). Furthermore. Shang et al. recognized that RNCR3 expression in MDSCs is upregulated by inflammatory and tumor associated factors. In the TME, the expression of RNCR3 was up-regulated in MDSC. RNCR3 may function as one ceRNA to upregulate the expression of Arg-1 and iNOS on MDSCs to enhance the roles of these MDSCs through sponge mir-185-5p which binds to CHOP to upregulate CHOP expression [104]. Therefore, those results suggest that RNCR3/miR-185-5p/Chop may strengthen suppressive roles of MDSCs in the TME.

## Concluding Remarks and Prospect

MDSCs, as immunosuppressive cells, seriously affect the progression, invasion and metastasis of tumors, and may be used as potential targets for tumor immunotherapy. The regulatory mechanism of tumor MDSCs has been widely investigated by us and other scientists (14, 92–95). Increasing evidence demonstrated that ncRNAs, especially miRNA and lncRNAs, played the key roles in the regulation of tumor MDSCs in the TME. Here we review that MiRNA/lncRNAs regulate the biological status and functional activity of tumor MDSCs through different regulatory mechanisms. Moreover, we discuss how both exosomal miRNAs/lncRNAs and the interaction of miRNAs/lncRNAs modulate tumor MDSCs. However, In the TME, the regulation of miRNAs/lncRNA on MDSCs is affected. It remains to be explored how those dysregulated miRNAs/lncRNA are combined in the TME to act on tumor MDSCs through tumor-related signaling pathway. In addition, the regulation of miRNA/lncRNAs on MDSCs provided opportunities and challenges for targeting MDSCs immunotherapy. Moreover, these functional data of miRNA/lncRNA on tumor MDSCs are gained from animal studies, there are a few data from human patients with cancer. Thus, miRNAs/lncRNAs application for tumor MDSCs in clinical patients with cancer need be further clarified. In summary, the interaction of dysregulated miRNAs/lncRNA on tumor MDSCs with transcription factors, cofactors and chromatin modifiers may target specific signals to treat tumor MDSCs in the TME, providing novel strategies for cancer treatment.

## Author Contributions

XL and SZ wrote, reviewed, and revised manuscript, figures and tables.HS prepared the figures and tables. HL and MY reviewed and revised the manuscript. YS revised the manuscript. PQ wrote, reviewed, and revised manuscript, figures and tables. All authors contributed to the article and approved the submitted version.

## Funding

This work was supported by the National Natural Science Foundation of China (Grant No. 81572868) and Science Foundation of Shandong (Grant No. ZR2018LC012).

## Conflict of Interest

The authors declare that the research was conducted in the absence of any commercial or financial relationships that could be construed as a potential conflict of interest.

## Publisher’s Note

All claims expressed in this article are solely those of the authors and do not necessarily represent those of their affiliated organizations, or those of the publisher, the editors and the reviewers. Any product that may be evaluated in this article, or claim that may be made by its manufacturer, is not guaranteed or endorsed by the publisher.

## Abbreviations

MDSCs, Myeloid-derived suppressor cells; TME, Tumor microenvironment; TGF-β, The transforming growth factor-β; IL-1β, The cytokine interleukin-1β; IFN-γ, Interferon-γ; VEGFA, vascular endothelial growth factor A; C/EBP, CCAAT/enhancer binding protein; CXCR2,C-X-C motif chemokine receptor 2; PTEN, Phosphatase and tensin homolog; SHIP-1, Src Homology 2-containing Inositol Phosphatase-1; YAP, Yes-associated protein; ASH2L, Absent small or homeotic-like; MLL1, Mixed lineage leukemia 1; CCL2, C-C motif chemokine ligand 2; MEF2C, Myocyte enhancer factor 2C; RaP1B, Ras-related protein Rap1B; MUC1, Transmembrane glycoprotein Mucin 1; IRF8, Interferon regulatory factor 8; NLRP3,NACHT; LRR; and PYD domains-containing protein 3; NFIA, Nuclear factor I A; AML1, Acute myeloid leukemia 1; IGF1, Insulin-like growth factor I; FOG2, Friend of Gata 2; PDCD4, Programmed cell death 4; SOCS3, Suppressor of cytokine signalling-3; PIAS3, Protein inhibitor of activated STAT3; SNAI1, Snail family transcriptional repressor 1; Twist1, Twist-related protein 1; ZEB1, Zinc finger E-box binding homeobox 1; LAMB3, Laminin subunit beta-3; CHOP, C/EBP Homologous Protein.
